# Integrated Immune, Epithelial and Lipid Pathways in NSAID‐Exacerbated Respiratory Disease

**DOI:** 10.1002/clt2.70182

**Published:** 2026-08-03

**Authors:** Piotr Szatkowski, Lucyna Mastalerz

**Affiliations:** ^1^ 2nd Department of Internal Medicine Jagiellonian University Medical College Krakow Poland

**Keywords:** biomarkers, effector cells, NSAID‐exacerbated respiratory disease

## Abstract

NSAID‐exacerbated respiratory disease (N‐ERD) is a chronic inflammatory disorder characterized by asthma, chronic rhinosinusitis with nasal polyps and respiratory reactions to cyclooxygenase‐1 inhibiting nonsteroidal anti‐inflammatory drugs (NSAID). Its pathogenesis involves dysregulated arachidonic acid metabolism, epithelial barrier dysfunction and activation of multiple immune cell types, including eosinophils, mast cells, macrophages, basophils, and innate lymphoid cells. Recent genomic and transcriptomic analyses have revealed variants in genes linked to epithelial integrity and cellular interactions, metabolic and epigenetic reprogramming of monocyte‐derived macrophages that promotes persistent proinflammatory activity. Emerging biomarkers, such as 15‐oxo‐eicosatetraenoic acid (15‐oxo‐ETE), acylcarnitines, apolipoprotein E, oncostatin M, surfactant protein D, retinoic acid and glial cell line‐derived neurotrophic factor provide novel mechanistic insights. A recently proposed Aspirin Hypersensitivity Diagnostic Index, integrating urinary leukotriene E4 (LTE4) and 15‐oxo‐ETE with sinus computed tomography (CT) scoring, may offer a practical noninvasive diagnostic tool. Inflammatory endotyping highlights substantial heterogeneity in N‐ERD, encompassing T2‐dominant, T1‐, T3‐related and neutrophilic inflammatory patterns, which may coexist or vary across airway compartments and influence clinical presentation and treatment response. As therapeutic strategies evolve, biologic agents targeting T2 pathways are becoming increasingly central, while aspirin therapy after desensitization may be reserved for selected patients. This review aims to summarize current mechanistic insights into the immune, epithelial, and lipid pathways involved in N‐ERD, with a particular focus on emerging biomarkers and inflammatory endotypes relevant to diagnosis and personalized therapy.

## 1 Introduction

NSAID‐exacerbated respiratory disease (N‐ERD) is clinically characterized by asthma, chronic rhinosinusitis with nasal polyposis (CRSwNP), and respiratory reactions to aspirin and other non‐steroidal anti‐inflammatory drugs (NSAIDs) that inhibit cyclooxygenase‐1 (COX‐1) [1, 2, 3]. Approximately 10% of adult patients with asthma are diagnosed with N‐ERD [1, 2, 3]. The onset and severity of symptoms are time‐dependent and manifest as upper and/or lower airway symptoms. Importantly, the initial inflammatory process begins and persists independently of exposure to NSAIDs [4, 5].

Airway inflammation is characterized by a potent type 2 immune response with activation of multiple effector cells, including eosinophils, mast cells, macrophages, basophils, innate lymphoid cell 2 (ILC2), epithelial cells, neutrophils, among others, as well as by disturbances in arachidonic acid (AA) metabolism [3, 6, 7]. Lipid mediators, including cysteinyl leukotrienes (cys‐LTs) and pro‐inflammatory prostaglandin D2 (PGD2), which are central to the pathogenesis of NSAID hypersensitivity, are potent ILC2 activators, which rapidly and robustly induce the production of T2 profile cytokines and other cytokines involved in broader inflammatory pathways [2, 3, 6].

This review summarizes the latest genomic, transcriptomic, molecular, and immunological insights into the pathogenesis of N‐ERD, including the roles of effector cells [3, 8, 9, 10, 11], as well as notable epithelial barrier [12, 13, 14, 15, 16], dysregulation of lipid and inflammatory mediators [3, 17, 18, 19, 20, 21]. It also discusses future directions in the diagnosis and treatment of NSAID hypersensitivity. Finally, we address whether a single biomarker or even a complex biomarker profile can sufficiently explain the mechanisms underlying asthmatic inflammation in patients with NSAID hypersensitivity.

## 2 Genetic Variants

Whole‐exome sequencing (WES) is used to detect rare genetic variants within all coding regions of an individual's genome. Exome sequencing offers the opportunity to detect rare protein‐coding variants causing monogenic susceptibility to a disease. Studies using WES in patients with N‐ERD have shown that approximately 5% exhibited monogenic susceptibility linked to pathogenic variants in the filaggrin (*FLG*) gene [13]. Additionally, variants were identified in genes related to epithelial integrity (e.g., desmoglein 3 (*DSG3*), dynein axonemal heavy chain 9 (*DNAH9*), collagen type VII alpha 1 chain (*COL7A1*), collagen type XVII alpha 1 chain (*COL17A1*)), and genes involved in cellular interactions (chromodomain helicase DNA binding protein‐7 (*CHD7*), TSC complex subunit 2/tuberous sclerosis‐2 protein (*TSC2*), P‐selectin (*SELP*), and platelet‐derived growth factor receptor‐alpha (*PDGFRA*)) [13]. These findings suggest that additional genetic factors may contribute to the development of N‐ERD. Exomiser, a phenotype‐driven variant prioritization software tool, may become a valuable approach for the clinical evaluation of genetic associations in the future [13]. Genetic variants related to epithelial integrity suggest a contributory role of barrier dysfunction in N‐ERD, although their clinical utility remains limited. Notably, genome‐wide association studies (GWAS) provide a broader perspective on N‐ERD susceptibility by capturing the polygenic and complex nature of the disease, including contributions from common variants beyond the scope of rare coding changes identified by WES.

## 3 Eosinophil Counts

The peripheral blood eosinophil count is usually higher in patients with N‐ERD compared with those in aspirin‐tolerant asthmatics (ATA) [8, 12, 22, 23]. However, this easily accessible laboratory biomarker lacks specificity. Interleukin‐5 (IL‐5) is a major cytokine involved in the regulation of peripheral blood eosinophil counts, as well as in eosinophil activation and migration into tissues [24]. Notably, IL‐5 serum concentrations do not differ between asthmatics with and without NSAID hypersensitivity [24]. Moreover, the effects of IL‐5 on cys‐LTs production are tissue‐specific, affecting cys‐LTs levels in the bronchi, rather than in the blood [24]. In addition, blood eosinophil counts have been reported to correlate only weakly with eosinophil counts in sputum [9, 25, 26], bronchoalveolar lavage fluid [27], nasal lavage [27] and airway tissue [28]. Interestingly, a significant increase in circulating and nasal polyps CD62L^low^ eosinophils has been observed in patients with severe asthma, which frequently accompanies NSAID hypersensitivity, compared to those with non‐severe asthma [29, 30]. It is therefore plausible that eosinophils are effector cells whose activation can be documented during a hypersensitivity reaction to NSAIDs. Consistently, a decrease in blood [6] and sputum [9] eosinophil counts has been observed during bronchospasm inducing by aspirin. However, during long‐term high dose aspirin therapy after aspirin desensitization (ATAD) peripheral blood eosinophilia either increases [20, 21, 31, 32, 33] or remains stable [34] while sputum eosinophil counts decrease [31]. Cases of hypereosinophilia (> 1500 cells/μL in blood) associated with ATAD have been reported in N‐ERD [32].

Notably, eosinophils collected from the respiratory tract in N‐ERD have additional properties, such as constitutive expression of leukotriene C4 synthase (*LTC4S*) leading to chronic release of cys‐LTs, production of interferon *γ* (INFγ) and PGD2, and overall sensitivity to aspirin, which stimulates mediator release in an aspirin dose‐dependent manner [20, 27, 35, 36, 37, 38]. Collectively, eosinophil counts and activation markers reflect ongoing T2 inflammation in N‐ERD but lack specificity as standalone diagnostic biomarkers.

## 4 Mast Cell

Aspirin‐induced bronchoconstriction is thought to involve mast cell activation resulting from the loss of locally produced anti‐inflammatory PGE_2_, which normally acts to stabilize mast cells [17, 39]. In the airways, PGE2 is produced mainly by epithelial cells and macrophages thereby contributing to local mast cell regulation [3]. Additionally, PGE2 limits Th2 differentiation and supports regulatory T‐cell expansion, promoting resolution of T2 inflammation [40]. Another important and still poorly understood aspect in the pathogenesis of N‐ERD is the fact that mast cells stimulated in vitro by alarmin interleukin 33 (IL‐33) release leukotriene C4 (LTC4), which initiates a feed‐forward loop by activating platelets [41]. Activated platelets further enhance LTC4 production by mast cells and promote the release of key allergic mediators, including PGD2 and histamine [41]. Neutralization of IL‐33 prevents the aspirin‐induced elevation of cys‐LTs and CXCL7, a marker of platelet activation, in the bronchoalveolar lavage fluid (BALF) of N‐ERD‐like mice [41]. In patients with severe asthma, BALF concentrations of PGD2 showed a strong correlation with both CXCL7 and mast cell tryptase levels [41]. Mast cells as a potential major source of leukotriene and prostaglandin in sinus tissue and scRNA‐seq of nasal polyps (NPs) from N‐ERD revealed that mast cells were the predominant cell type in N‐ERD expressing arachidonate 5‐lipoxygenase (*ALOX5*), arachidonate 5‐lipoxygenase activating protein *(ALOX5AP),* leukotriene C4 synthase *(LTC4S)*, and gamma‐glutamyltransferase 1 (*GGT1*) genes encoding enzymes involved in cys‐LTs metabolism [42]. This suggests that post‐transcriptional mechanisms, rather than gene‐expression differences, may contribute to the characteristic AA metabolism imbalance associated with mast cells in N‐ERD [42]. Mast cell derived mediators, particularly cys‐LTs and PGD2, highlight mast cells as key amplifiers of airway inflammation in N‐ERD and potential indicators of disease activity.

## 5 Mast Cell and Epithelial Cell Interactions

It was concluded that interactions between mast cells and nasal epithelial cells, leading to the local synthesis of 15‐oxo‐eicosatetraenoic acid (15‐oxo‐ETE), may contribute to the dysregulation of AA metabolism via the 15‐lipoxygenase (15‐LOX) pathway in N‐ERD [42]. Indeed, the *ALOX15* gene, which encodes 15‐LOX, was significantly upregulated in nasal polyps from patients with N‐ERD compared with those nasal polyps from patients with CRSwNP without NSAID hypersensitivity or from healthy controls (HCs) [42]. *ALOX15* was expressed predominantly by nasal epithelial cells, and its expression levels correlated significantly with clinical asthma severity and radiographic sinus disease [42]. Notably, hydroxyprostaglandin dehydrogenase (*HPGD*), which is required for 15‐oxo‐ETE synthesis, was predominantly expressed in mast cells and eosinophils, and localized near 15‐LOX‐expressing epithelium in nasal polyps from patients with N‐ERD [42].

## 6 Monocyte and Macrophage

Monocytes, as well as macrophages, from patients with N‐ERD exhibit global DNA hypomethylation, disrupted metabolic profiles and elevated chemokine expression, indicative of sustained proinflammatory activation [10]. In vitro experiments revealed that blood monocytes isolated from patients with N‐ERD compared with those from HCs, when exposed to a lung‐adapted cytokine milieu (TGF‐β1 and GM‐CSF) showed 86 downregulated and 19 upregulated genes relative to HCs [10].

Macrophages are major cellular players in metabolic reprogramming and represent an important source as well as a cellular target of fatty acid metabolites during type 2 inflammation [10, 43, 44, 45]. It was documented that an oral aspirin challenge reduced sputum macrophage percentages, regardless of the occurrence of bronchospasm and NSAID hypersensitivity in asthmatic patients [9]. However, only in patients with N‐ERD macrophages have been shown to undergo metabolic and epigenetic changes, leading to a persistent proinflammatory state. These macrophages produce higher levels of pro‐inflammatory acylcarnitine, proinflammatory AA metabolites, cytokines and chemokines compared with healthy macrophages [10, 11, 42, 43]. Indeed, the studies revealed higher levels of acylcarnitine to be associated with increased fatty acid oxidation and airway inflammation in murine model of asthma [46]. The elevated levels of acylcarnitine in macrophages and body fluids of patients with N‐ERD suggest a role of this metabolite in type 2 inflammation [10]. Indeed, most acylcarnitine species were found to be elevated in alveolar monocyte‐derived macrophages (aMDMs) from patients with N‐ERD compared with aMDMs from HCs [10].

Recent research has highlighted the role of 15‐oxo‐ETE, a metabolite produced by macrophages and monocytes, eosinophils, mast cells, and epithelial cells [8], which may play a significant role in the pathogenesis of N‐ERD [3]. Indeed, expression of *ALOX15* and proinflammatory M2‐associated markers (*CCL17* and *TGM2*) tended to be increased in patients with N‐ERD compared with those from HCs [10, 42]. Furthermore, a high capacity to generate proinflammatory 5‐lipoxygenase (5‐LOX) products by macrophages and M2 activation is characteristic for N‐ERD [10, 43].

Since macrophages are a major source of Apolipoprotein E (ApoE), which protects against airway inflammation, its deficiency may promote chronic type 2 inflammation. *ApoE* knockdown in macrophages increases CXCL7 production and may contribute to oxidative cell death (ferroptosis) associated with type 2 inflammation [11]. Restoring *ApoE* could help restore epithelial barrier integrity and macrophage function in N‐ERD [11].

M2 macrophages orchestrate Th2‐dominant immune responses via the recruitment of Th2 lymphocytes and eosinophils to the airways [45]. Serine peptidase inhibitor, clade B, member 10 (SERPINB10) promotes M2 polarization, and is related to eosinophilic inflammation in asthma [45]. It was suspected, that studying macrophage phenotypes and the cytokine profiles they release could provide valuable insights into their role in N‐ERD and the use of biological therapies in these patients [45]. Macrophages in N‐ERD are best viewed as integrative and amplifying cells that translate epithelial and lipid‐mediated signals into sustained inflammatory activity, rather than as primary initiating drivers.

## 7 Basophil

Patients with N‐ERD exhibit increased numbers of basophils in NPs and in peripheral blood [47]. Analysis of NPs tissue revealed that basophil counts were positively correlated with eosinophil numbers and, to a lesser extent, with mast cell numbers [47]. While eosinophils were significantly increased in nasal polyps from patients with N‐ERD, mast cells showed only a nonsignificant trend toward elevation [47]. In addition, circulating basophil counts were positively correlated with blood eosinophil counts in N‐ERD [47].

Furthermore, analysis of basophil activation markers demonstrated an increased proportion of highly activated basophils (CD63^+^/CD203c^+^) in NPs from patients with N‐ERD compared with CRSwNP, accompanied by reduced expression of the granule marker 2D7 [47]. The number of active basophils (CD63^high^ and 2D7^low^) in NPs in N‐ERD was correlated with higher Lund‐Mackay score, but negatively correlated with FEV1 [47]. Thus, active basophils in NPs are directly connected to lower lung function and more severe lower airway disease [47]. Basophil activation and tissue accumulation may reflect disease severity and upper‐airway involvement, suggesting a supportive role in N‐ERD.

## 8 Innate Lymphoid Cells

Innate lymphoid cells (ILCs) represent a diverse group of innate immune cells that, despite lacking the antigen‐specific receptors characteristic of B and T lymphocytes, can mirror the functional profiles of Th1, Th2, and Th3 subsets of T‐helper cells [48]. This functional flexibility allows them to rapidly respond to epithelial‐derived signals and shape airway inflammation in diseases such as N‐ERD. Among these populations, ILC2 cells have received particular attention in the context of N‐ERD. They express receptors for cysteinyl leukotrienes and prostaglandin D_2_ (PGD_2_), making them highly responsive to the dysregulated AA‐derived mediators that are a hallmark of this condition [49]. Eicosanoids binding to these receptors function as potent chemoattractants for ILC2, while alarmin cytokines TSLP, IL‐25, and IL‐33 serve as their major activating stimuli [7]. Once activated, ILC2 cells release substantial quantities of IL‐4, IL‐5, and IL‐13, thereby amplifying type‐2 driven inflammation [7].

Importantly, clinical challenge studies have shown that during aspirin exposure, ILC2 cells accumulate prominently within the nasal mucosa, where they likely contribute to the local surge in inflammatory mediators [7, 50]. Interestingly, this accumulation does not occur in induced sputum during aspirin‐induced bronchospasm, suggesting a striking compartmentalization of the ILC2 response between upper and lower airways [6]. This discrepancy highlights the possibility that tissue‐specific microenvironments modulate ILC2 behavior a concept that may help explain the clinical heterogeneity of N‐ERD.

**FIGURE 1:**
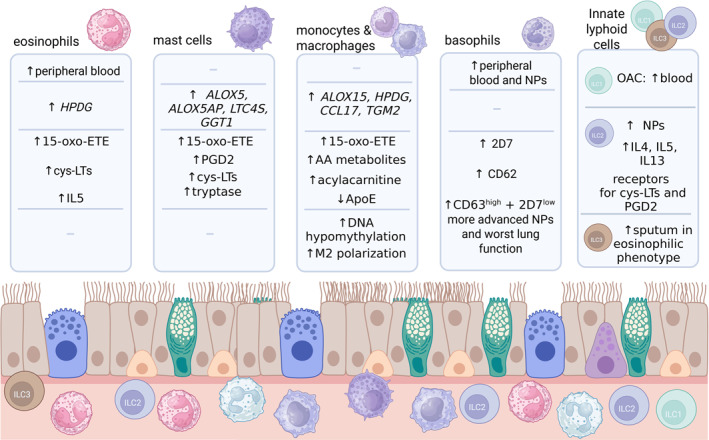
Cells involved in pathogenesis of N‐ERD. Alterations in eosinophils, mast cells, monocytes/macrophages, basophils and innate lymphoid cells contribute to dysregulated lipid mediator metabolism, immune activation and airway inflammation. Key cell‐specific mediators, enzymes and activation markers associated with disease mechanisms are shown. Figure was prepared with BioRender.com. 15‐oxo‐ETE, 15‐oxo‐eicosatetraenoic acid; 2D7, basophil secretory granule marker; ALOX15, arachidonate 15‐lipoxygenase; ALOX5, arachidonate 5‐lipoxygenase; ALOX5AP, arachidonate 5‐lipoxygenase‐activating protein; ApoE, apolipoprotein E; CCL17, chemokine (C‐C motif) ligand 17; CD62, L‐selectin; CD63, degranulation marker; cys‐LTs, cysteinyl leukotrienes; GGT1, gamma‐glutamyltransferase 1; HPDG, 15‐hydroxyprostaglandin dehydrogenase; IL‐13, interleukin 13; IL‐4, interleukin 4; IL‐5, interleukin 5; ILC1, innate lymphoid cell type 1; ILC2, innate lymphoid cell type 2; ILC3, innate lymphoid cell type 3; LTC4S, leukotriene C4 synthase; NPs, nasal polyps; OAC, oral aspirin challenge; PGD_2_, prostaglandin D_2_; TGM2, transglutaminase 2.

Further evidence points to a lipid‐mediator‐dependent regulation of ILC2 trafficking. It was suggested that lower serum levels of 15‐hydroxyeicosatetraenoic acid (15‐HETE) and higher 19, 20‐dihydroxy‐4Z,7Z,10Z,13Z,16Z‐docosapentaenoic acid (19,20‐diHDPA) predict a greater accumulation of airway ILC2 in the nasal mucosa [50]. This observation supports the idea that ILC2 responses are tightly intertwined with the dysregulated lipid metabolism characteristic of N‐ERD. Furthermore, N‐ERD patients showed a significant increase in blood ILC1s during aspirin‐induced bronchospasm, while no changes in ILC2 in the lower airway were observed [6]. Additionally, patients with an eosinophilic asthma phenotype showed increased proportions of ILC3 cells in sputum compared with individuals with non‐eosinophilic asthma [6]. This finding suggests that, at least in a subset of patients, pathways associated with Th3‐related inflammation may also be active. Taken together, these observations underscore an emerging concept: N‐ERD encompasses multiple overlapping inflammatory programs and ILCs spanning ILC1, ILC2, and ILC3 subsets likely contribute to its endotype diversity, see Figure 1. Alterations in ILC subsets, particularly ILC2 and compartment‐specific ILC responses, underscore the heterogeneity of immune endotypes in N‐ERD and may inform inflammatory stratification.

## 9 Impact of Extracellular Vesicles on Macrophages

Extracellular vesicles (EVs) are membrane‐bound particles involved in intercellular communication and immune regulation [14, 51]. In N‐ERD, EVs detected in nasal lining fluid show markers of macrophage and epithelial origin and display associations with cysteinyl leukotriene levels and mast cell activity [14]. Experimental studies indicate that EVs derived from induced sputum may modulate macrophage cytokine and prostanoid responses and influence macrophage polarization toward an M2‐like phenotype, potentially mediated by specific miRNA profiles [51]. However, the role of EVs in N‐ERD pathophysiology remains incompletely characterized and requires further validation in patient‐based studies.

## 10 Proposed Hierarchical Model of Pathogenic Axes in N‐ERD

Based on current evidence, we propose a hierarchical model in which epithelial dysfunction represents the primary upstream driver of N‐ERD pathogenesis. Impaired epithelial barrier integrity and type‐2 driven epithelial activation promote upregulation of the 15‐lipoxygenase‐1 (15‐LOX‐1) pathway, leading to increased local generation of 15‐oxo‐ETE and creating a lipid mediator milieu that facilitates downstream immune activation.

Within this context, IL‐33 dependent mast cell–platelet interactions act as secondary amplifying mechanisms, particularly during acute NSAID exposure, driving rapid increases in cysteinyl leukotrienes and PGD_2_ and contributing to clinical reactions. In parallel, macrophage metabolic reprogramming characterized by increased acylcarnitine accumulation represents a disease‐perpetuating axis, sustaining chronic inflammation, endotype heterogeneity, and treatment variability independently of acute drug challenge.

**FIGURE 2:**
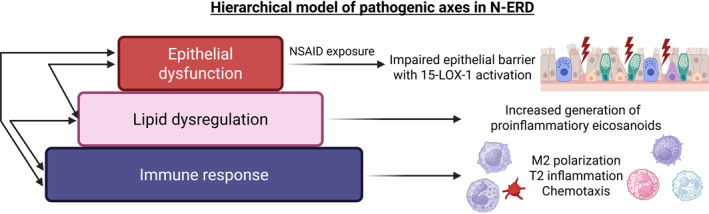
Model of pathogenic axes in N‐ERD. The model illustrates epithelial dysfunction as an upstream event driving lipid mediator dysregulation and downstream immune activation, with bidirectional interactions forming self‐amplifying feedback loops. 15‐LOX‐1, 15‐lipoxygenase‐1; M2, alternatively activated macrophages; T2, type 2 inflammation.

This framework prioritizes epithelial‐lipid dysregulation as the initiating event while recognizing that downstream immune axes remain highly interconnected and mutually reinforcing rather than strictly linear, see Figure 2.

## 11 Novel Biomarkers

*15‐oxo‐eicosatetraenoic acid* is the terminal metabolite of the AA pathway mediated by 15‐lipoxygenase, encoded by *ALOX15*, and is strongly upregulated in the epithelium of in T2‐high asthma and nasal polyps in N‐ERD [15, 16, 42]. Stimulation of the airway epithelium by IL‐4 and IL‐13 increases basal cell populations and induces upregulation of thymic stromal lymphopoietin (*TSLP*), *IL‐33*, as well as *ALOX15* [16]. Some variants in epithelial genes *ALOX15* and *IL33* were described as associated with an increased risk of nasal polyposis [16].

Basal levels of 15‐oxo‐ETE are markedly higher in plasma and NPs in patients with N‐ERD [8, 22, 42]. Cluster analysis revealed that higher plasma and lower urinary levels of 15‐oxo‐ETE are associated with more severe asthma and worse asthma control in patients with N‐ERD, especially in older individuals and those with a higher body mass index [8]. On the other hand, local neo‐synthesis of 15‐oxo‐ETE in induced sputum supernatant was significantly decreased during the bronchospasm induced by aspirin [6, 23]. Thus, aspirin‐induced bronchospasm inhibits the local generation of 15‐oxo‐ETE.

Apparently, AA metabolism via the 15‐LOX pathway plays a role in the pathogenesis of N‐ERD. It was proposed that the *Aspirin Hypersensitivity Diagnostic Index* (AHDI) be based on urinary LTE4 to 15‐oxo‐ETE excretion corrected for sex and the Lund‐Mackay score of chronic rhinosinusitis to identify N‐ERD [22]. AHDI was characterized by an area under the ROC curve of 0.889, sensitivity of 81.97%, specificity of 87.23%, and accuracy of 86.87% [22]. Therefore, we suggest that a single urine sample to examine urinary 15‐oxo‐ETE and LTE4 levels, together with the CT scan of the paranasal sinuses, can distinguish N‐ERD in a patient with asthma [22]. AHDI seems feasible in daily clinical practice but requires external validation.

*Apolipoprotein E (ApoE)* deficiency in the N‐ERD nasal mucosa affects the crosstalk and inflammatory activation of macrophages and epithelial cells [11]. Nasal scrapings from patients with N‐ERD exhibited decreased *APOE* expression compared with that of the nasal mucosa of HCs, but *APOE* was inherently low in epithelial cells. In addition, myeloid cells expressed highly abundant *APOE*, which was reduced in monocyte‐derived macrophages from patients with N‐ERD [11]. Moreover, oxidized arachidonyl‐phosphatidylethanolamine accumulated in *APOE*‐knockdown macrophages, and ApoE protected macrophages from ferroptotic cell death [11]. Interestingly, in the context of AA eicosanoids, exogenous ApoE reduced PGE2 levels, whereas *APOE* knockdown tended to increase the PGE2 pathway, suggesting that reduced ApoE is not responsible for aberrant PGE2 metabolism in N‐ERD [11].

*Oncostatin M* (OSM), the IL‐6 family‐related cytokine oncostatin M, and IL‐6 have been shown to promote mucosal epithelial barrier dysfunction and can promote epithelial barrier permeability [52]. These findings reflect a complex multicellular axis involving IL‐4Rα‐expressing innate tissue resident mast cells and macrophages as sources of OSM, with mast cells providing CSF‐1 and CSF‐2 to promote macrophage survival, while IL‐4/IL‐13 signaling has been associated with OSM production, as well as OSM generation by neutrophils. Additionally, it was reported that fibroblasts generate IL‐6 in response to both OSM and IL‐13, with an additive effect [52]. These findings support the conclusion that the reduction in OSM following dupilumab treatment is likely due to direct and indirect inhibitory effects of blocking IL‐4Rα on mast cells and monocytes/macrophages, and that IL‐4Rα inhibition can both directly and indirectly affect fibroblast IL‐6 production [52].

*Surfactant protein D (SPD)* is a member of the collection family that contributes to host defense at the airway epithelium. The serum SPD level was significantly lower in N‐ERD compared with ATA patients and negatively correlated with the fall in FEV_1_ (%) and urinary level of LTE_4_ after aspirin challenge [53, 54]. The decreased level of SPD in N‐ERD was associated with airway inflammation/remodeling under the eosinophilic condition, suggesting that modulation of SPD may provide a potential benefit in N‐ERD [53].

*Retinoic acid (RA)* is necessary for maintaining epithelial function and is a well‐known inducer of tissue plasminogen activator (tPA) in endothelial cells and it was reported that RA, vitamin A, D‐dimer levels and expression of *tPA* were lower in NPs from patients with N‐ERD compared with ATA [55]. These findings likely reflect a reduced fibrinolytic capacity in N‐ERD‐associated polyps, even when compared with NPs from ATA [55].

*The glial cell line‐derived neurotrophic factor (GDNF)* gene encodes a secreted TGF‐β related ligand, and its expression in nasal secretions was found to be elevated in N‐ERD [56]. Notably, only in N‐ERD was *GDNF* expression positively correlated with the Total Polyp Score [56]. *GDNF* was shown to promote a proinflammatory cytokine profile, its involvement in airway remodeling and promotion of type 2 inflammation suggests a link to impaired barrier function causative of CRSwNP, especially in N‐ERD [56].

**FIGURE 3:**
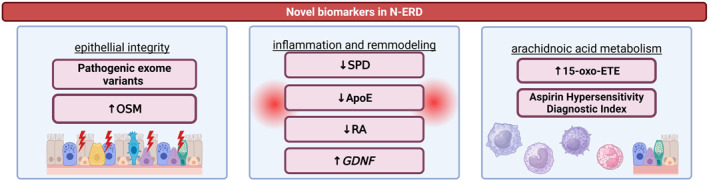
Novel biomarkers in N‐ERD. Summary of selected emerging biomarkers related to epithelial integrity, inflammation, remodeling and AA metabolism in N‐ERD. Figure was prepared with BioRender.com. 15‐oxo‐ETE, 15‐oxo‐eicosatetraenoic acid; ApoE, Apolipoprotein E; *GDNF*, glial cell line‐derived neurotrophic factor; OSM, oncostatin M; RA, retinoic acid; SPD, surfactant D protein.

In summary, mentioned biomarkers beyond AHDI may have clinical utility in N‐ERD primarily for disease stratification and therapeutic decision‐making rather than for standalone diagnosis. Blood eosinophils, sputum cellular profiles and selected lipid or epithelial‐derived markers may help identify dominant inflammatory pathways and support personalized management, see Figure 3 and Table 1.

**TABLE 1:** Summary of selected biomarkers associated with N‐ERD.

Biomarker	Source/compartment	Associated pathway	Proposed clinical relevance	Level of evidence
Blood eosinophils	Peripheral blood	Type 2 inflammation	Phenotyping, therapy selection	Clinical
Sputum cell profiles	Lower airways	Mixed inflammatory pathways	Endotype stratification	Clinical
Extracellular vesicles	Airway fluids	Intercellular signaling	Exploratory biomarkers	Experimental
LTE4	Urine, serum	AA metabolism	Diagnostic support, disease activity	Clinical studies
15‐oxo‐ETE	Plasma, NPs	15‐LOX‐1 pathway	Disease severity, endotyping	Clinical/experimental
AHDI	Urine (LTE4, 15‐oxo‐ETE) + sinus CT	AA metabolism + airway remodeling	Composite, non‐invasive diagnostic tool	Clinical (single‐center), requires external validation
ApoE	Macrophages, epithelium	Lipid handling, barrier function	Disease modulation	Experimental
OSM	Mast cells, macrophages	Barrier dysfunction	Disease severity	Experimental/clinical
SPD	Airway epithelium	Innate immunity	Airway inflammation	Clinical
Acylcarnitine	Macrophages, plasma	Metabolic reprogramming	Endotype characterization	Experimental
RA	NPs	Barrier integrity, fibrinolysis	Upper airway remodeling, polyp formation	Experimental
GDNF	Nasal secretions	Epithelial dysfunction, tissue remodeling	Disease severity, nasal polyp burden	Experimental

Abbreviations: 15‐LOX‐1, 15‐lipoxygenase‐1; 15‐oxo‐ETE, 15‐oxo‐eicosatetraenoic acid; AA, arachidonic acid; AHDI, Aspirin Hypersensitivity Diagnostic Index; ApoE, apolipoprotein E; CT, computed tomography; GDNF, glial cell line‐derived neurotrophic factor; LTE4, leukotriene E4; N‐ERD, NSAID‐exacerbated respiratory disease; NP(s), nasal polyp(s); OSM, oncostatin M; RA, retinoic acid; SPD, surfactant protein D.

## 12 Endotypes and Phenotypes

While N‐ERD appears relatively homogeneous with respect to lipid mediator profiles, it shows considerable variability in type 1, type 2, and type 3 cytokine levels. This heterogeneity may influence therapeutic responses to treatments targeting type 2 inflammation. Overall, patients with N‐ERD exhibit a diverse inflammatory landscape characterized by fluctuating levels of these cytokines. Current findings suggest the presence of nonclassical spectrum T2 inflammation in about half the patients with N‐ERD [57, 58, 59, 60].

Further studies are needed to clarify the collaboration of T2 inflammation with M2 macrophages, neutrophils, and fibroblasts, as well as the downregulation of innate host defense mechanisms in patients with N‐ERD. Although the frequency of single T2 endotype was lower in patients with N‐ERD than in those with NSAID‐tolerant CRSwNP, it did not reach statistical significance [61]. Recently, this mechanism was described in patients with high sinus CT and NP scores [61]. Moreover, it was suggested that T3 and neutrophilic inflammation are associated with each other, but neutrophilic inflammation can also be present in any inflammatory endotype, even in the absence of T3 [61]. Thus, a neutrophil variant (Vneut) was proposed as a new endotype distinct from the T3 endotype. The marker of the Vneut (*FCGR3B*) endotype was elevated in N‐ERD‐NPs compared with NSAID‐tolerant NPs [61]. Indeed, the effects of non‐T2 inflammation in Th2 and ILC2 asthma with CRSwNP and NSAID hypersensitivity clinical presentations and their association with underlying molecular mechanisms require clarification. While N‐ERD is characterized by marked heterogeneity of inflammatory endotypes (T1/T2/T3/Vneut), these profiles reflect different upstream regulatory programs that converge on common downstream effector mechanisms. Thus, endotypic diversity and a unifying effector framework are not mutually exclusive but represent complementary aspects of disease pathogenesis.

Bronchial brushing endotypes in the lower airways of patients with N‐ERD were defined as (i) a T2 pattern having an average of 30‐fold greater expression of T2 inflammatory genes than in controls and (ii) a proinflammatory pattern having an average of 9‐fold greater expression of innate and proinflammatory/IL‐17A response genes than in controls. When this definition was used, 23% of patients with N‐ERD had a T2 endotype, 18% had a proinflammatory endotype, and 9% had a mixed proinflammatory endotypes, and 50% had neither endotype [12]. Moreover, an eosinophilic endotype defined as the presence of at least 2% eosinophils in BALF was observed in 54% of patients with N‐ERD whereas 14% had a neutrophilic endotype defined as the presence of at least 3% neutrophils. Another 32% of patients had a paucigranulocytic endotype defined as having neither an eosinophilic nor a neutrophilic endotype [12].

Interestingly, an analysis of inflammatory phenotype based on sputum cells from 133 patients with N‐ERD classified patients as eosinophilic (> 3% eosinophils and < 64% neutrophils), neutrophilic (> 64% neutrophils and < 3% eosinophils), mixed (> 3% eosinophils and > 60% neutrophils), and paucigranulocytic (< 3% eosinophils and < 64% neutrophils) [58]. When this classification was used, 46% of patients with N‐ERD had an eosinophilic inflammatory phenotype, 12% had a neutrophilic, 4% had a mixed eosinophilic and neutrophilic, and 38% had a paucigranulocytic [62]. Future studies are needed to refine which biomarkers are best at characterizing distinct inflammatory endotypes and how these endotypes may correspond with aspects of biological therapy.

**FIGURE 4:**
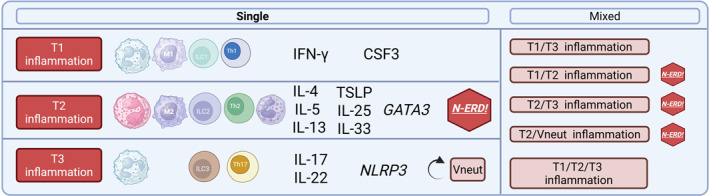
Endotypes in N‐ERD. Summary of single and mixed inflammatory endotypes in N‐ERD, defined by dominant cytokine profiles and associated immune cell involvement. Figure was prepared with BioRender.com. CSF3, colony‐stimulating factor 3; FCGR3B, Fc gamma receptor IIIb; *GATA3*, GATA‐binding protein 3; IFN‐γ, interferon gamma; IL‐4, interleukin‐4; IL‐5, interleukin‐5; IL‐13, interleukin‐13; IL‐17, interleukin‐17; IL‐22, interleukin‐22; IL‐25, interleukin‐25; IL‐33, interleukin‐33; ILC1, innate lymphoid cell type 1; ILC2, innate lymphoid cell type 2; ILC3, innate lymphoid cell type 3; M1, classically activated macrophage; M2, alternatively activated macrophage; *NLRP3*, NLR family pyrin domain containing 3; Th1, T‐helper 1 lymphocyte; Th2, T‐helper 2 lymphocyte; Th17, T‐helper 17 lymphocyte; TSLP, thymic stromal lymphopoietin; Vneut, neutrophilic variant endotype.

Supporting the presence of type 2 inflammation in N‐ERD, gene expression levels of *GATA3*, *IL‐4, IL‐5*, and *IL‐17* were significantly higher in nasal polyp tissue from N‐ERD patients compared to control group [63]. Expression of *GATA3* in sputum cells could be used as a useful biomarker for differentiating distinct severe asthma subtypes in patients with N‐ERD characterized by complex multicellular inflammatory involvement and one primarily driven by Th2 and/or ILC2 cells [64]. Clusters with low expression of *GATA3* in sputum were associated with increased PGD2 levels in induced sputum supernatant, tended to have a higher Lund‐Mackay score and elevated peripheral blood eosinophilia, and showed higher expression of genes related to the AA pathway compared to cluster with high expression of *GATA3* [64], see Figure 4.

## 13 Aspirin Therapy After Desensitization Versus Biologic Drugs

There are two main guidelines for the use of long‐term high‐dose aspirin therapy after aspirin desensitization (ATAD) in patients with N‐ERD [65, 66]. Numerous open and single double‐blind trials have demonstrated the beneficial clinical effects of ATAD in patients with N‐ERD [34, 67, 68, 69, 70, 71]. A prospective, double‐blind, randomized, cross‐over, placebo‐controlled study assessing the clinical effects caused by long term aspirin therapy is still lacking. ATAD may need reevaluation in the current era of biologic therapies. Several factors must be considered when choosing between ATAD, biologics, or a combination of both [65, 72]. Many experts agree that the primary benefit of ATAD is for the prevention of NPs regrowth and improvement in quality of life after debulking surgery, rather than for the reduction of existing polyp burden, improved asthma control, and increased FEV1 [65, 66]. The data published by A. Helevä et al. emphasize the small benefits of ATAD while highlighting the need to carefully consider the adverse effects of this therapy [70]. Clinical response to ATAD in N‐ERD appears heterogeneous, with worsening of respiratory symptoms associated with low baseline plasma 15‐HETE levels and Black or Latino ethnicity [33], whereas a favorable response has been linked to female sex, elevated blood eosinophil counts, low sputum neutrophil percentages, severe nasal symptoms, high *HPGD* expression, and low *PRG2* expression [38]. It seems that in the era of biological treatments, the time has come for long‐term aspirin therapy after aspirin desensitization should be recommended only for selected patients.

In large clinical trials of mepolizumab for CRSwNP, treatment responses were heterogeneous, with nearly half of patients reporting symptom worsening or no change, yet the therapy was approved for this indication, including N‐ERD [73]. In contrast, dupilumab has so far demonstrated greater efficacy in patients with N‐ERD than in ATA patients across several clinical outcomes [74]. Reported adverse events were generally mild to moderate, most commonly headache, injection‐site reactions, upper respiratory tract infections, and fatigue, with other events occurring infrequently [75].

## 14 In Conclusion

This review highlighted novel mechanistic findings contributing to the pathogenesis of N‐ERD. In this context, macrophages may also play an important role in N‐ERD pathogenesis. Indeed, compared with macrophages from healthy controls, alveolar‐like monocyte‐derived macrophages from patients with N‐ERD were more activated and had downregulated host defense–related genes [10]. Additionally, extracellular vesicles isolated from sputum of patients with N‐ERD were associated with increased production of inflammatory cytokines by cultured human bronchial epithelial cells in vitro as well as with the activation of M2 macrophages [10, 42]. The abnormally activated state of macrophages they identified in N‐ERD is notable for the increased production of cytokines and chemokines, and the increased release of proinflammatory lipids. The lipids that play a key role in macrophages' activation include lipids derived from the metabolism of AA, particularly leukotrienes produced by 5‐lipoxygenase and 15‐oxo‐ETE produced by 15‐lipoxygenase, as previously reported [3, 10, 43], but also acylcarnitine metabolites [43] regulating the crosstalk between macrophages and epithelial cells and contributing to ferroptosis during type 2 airway inflammation [11]. Reduced methylation of genes involved in fatty acid/acylcarnitine metabolism, and increased levels of acylcarnitine metabolites were found in the nasal fluid, sputum, and plasma of patients with N‐ERD [43]. Based on this evidence, we speculate that in the hypocellular phenotype of sputum, which contains the highest percentage of macrophages [10, 43] that can polarize to M2, biologics may be effective regardless of other T2 biomarkers. The newly described Vneut endotype requires further investigation, especially in the lower respiratory tract. Moreover, an important step forward in the in vitro diagnosis of NSAID hypersensitivity is the future validation of AHDI.

Several emerging biomarkers discussed in this review are currently supported by experimental or associative data and should be interpreted as exploratory rather than clinically validated markers. Future research should focus on the mechanistic validation of emerging pathways implicated in N‐ERD, including macrophage acylcarnitine metabolism, EVs‐mediated signaling. In addition, epithelial‐derived cytokine pathways (e.g., IL‐33, TSLP), mast cell‐platelet interactions, lipid mediator‐dependent macrophage, ILC activation represent promising targets for experimental and translational studies to define causality and therapeutic relevance.

The future of N‐ERD therapy will be biologics, including new potential targets such as a small molecules, extracellular vesicles [14, 51]. ATAD will be reserved for only a select few patients, making this treatment a historic one. Biologics are increasingly positioned as a central therapeutic option in severe N‐ERD.

## Author Contributions

**Piotr Szatkowski:** visualization, investigation, writing – original draft, writing – review and editing, data curation. **Lucyna Mastalerz:** conceptualization, investigation, writing – original draft, supervision, writing – review and editing, project administration.

## Funding

The authors have nothing to report.

## Ethics Statement

This review is a synthesis of previously published, publicly available scholarly articles and does not contain any original data. Consequently, the need for ethical approval from an institutional review board (IRB) or equivalent committee was considered not applicable. The authors confirm that all source material has been appropriately cited and referenced.

## Conflicts of Interest

The authors declare no conflicts of interest.

## Data Availability

The data that support the findings of this study are available from the corresponding author upon reasonable request.
